# Design of a complex virtual reality simulation to train finger motion for persons with hemiparesis: a proof of concept study

**DOI:** 10.1186/1743-0003-6-28

**Published:** 2009-07-17

**Authors:** Sergei V Adamovich, Gerard G Fluet, Abraham Mathai, Qinyin Qiu, Jeffrey Lewis, Alma S Merians

**Affiliations:** 1 New Jersey Institute of Technology, Department of Biomedical Engineering Newark, NJ, USA; 2 University of Medicine and Dentistry of New Jersey, Department of Rehabilitation and Movement Science, Newark, NJ, USA

## Abstract

**Background:**

Current neuroscience has identified rehabilitation approaches with the potential to stimulate adaptive changes in the brains of persons with hemiparesis. These approaches include, intensive task-oriented training, bimanual activities and balancing proximal and distal upper extremity interventions to reduce competition between these segments for neural territory.

**Methods:**

This paper describes the design and feasibility testing of a robotic/virtual environment system designed to train the hand and arm of persons with hemiparesis. The system employs a simulated piano that presents visual, auditory and tactile feedback comparable to an actual piano. Arm tracking allows patients to train both the arm and hand as a coordinated unit, emphasizing the integration of both transport and manipulation phases. The piano trainer includes songs and scales that can be performed with one or both hands. Adaptable haptic assistance is available for more involved subjects. An algorithm adjusts task difficulty in proportion to subject performance. A proof of concept study was performed on four subjects with upper extremity hemiparesis secondary to chronic stroke to establish: a) the safety and feasibility of this system and b) the concurrent validity of robotically measured kinematic and performance measures to behavioral measures of upper extremity function.

**Results:**

None of the subjects experienced adverse events or responses during or after training. As a group, the subjects improved in both performance time and key press accuracy. Three of the four subjects demonstrated improvements in fractionation, the ability to move each finger individually. Two subjects improved their aggregate time on the Jebsen Test of Hand Function and three of the four subjects improved in Wolf Motor Function Test aggregate time.

**Conclusion:**

The system designed in this paper has proven to be safe and feasible for the training of hand function for persons with hemiparesis. It features a flexible design that allows for the use and further study of adjustments in point of view, bilateral and unimanual treatment modes, adaptive training algorithms and haptically rendered collisions in the context of rehabilitation of the hemiparetic hand.

## Background

Stroke remains the leading cause of serious, long-term disability. With over 5.7 million stroke survivors in the United States [1], only five percent regain full upper extremity function, despite having had intensive therapy to address the disability [2]. While developing effective interventions to facilitate hand recovery is challenging, this is an important and needed aspect of rehabilitation. According to Manchke et al. [3], adaptive training paradigms that continually and interactively move the motor outcome closer and closer to the targeted skill are believed to be important to foster the formation of better organized motor skills. Computerized systems are well suited for accomplishing these goals. In particular, virtual-reality based simulations, allow for online adaptation and modification of task difficulty based on the participant s success rate and motor improvement.

This paper describes the Virtual Piano Trainer, a complex simulation, intended to train individual finger motion that provides realistic auditory and visual feedback of appropriate piano notes, sounds and music and combines hand movements with arm tracking. The VR system described in this paper allows us to manipulate the visual point of view when moving one s hands and manipulating objects. We present the virtual hands in a first person perspective because processing visual information gathered by looking down at one s own hand is easier and more intuitive than through the third person perspective (similar to looking in a mirror) The virtual piano trainer also provides auditory feedback in the form of music, which has specific benefits ascribed to it in the rehabilitation literature [4], and the VR literature [5]. Auditory feedback is both intrinsic and meaningful to the task of piano playing in this simulation.

Adding haptic feedback to auditory and visual feedback has been identified as an important adjunct to skill development particularly when the task difficulty is high[6] Several haptic devices designed for the hand provide tactile feedback during rehabilitation activities in virtual environments[6]. Other haptic devices use an extensor force on the fingers to inhibit the mass grasp pattern commonly seen in persons with stroke when performing activities [7-10]. The system we have developed is capable of providing both of these features

In the system that we have developed, we aid finger extension by integrating the commercially available haptic device, the CyberGrasp with the Virtual Piano Trainer into a single training system. The CyberGrasp is an exoskeleton device situated on the dorsum of the hand which allows for multiplanar arm motion while exerting an extensor force on each individual finger (Immersion, USA).

Recent literature suggests that balancing the training of both proximal and distal components of the upper extremity in order to minimize over-representation of the upper arm in re-organizing cortical territory [11]and facilitating inherent interlimb coordination through bilateral training might improve functional therapeutic outcomes for the arm and hand post-stroke. These concepts have led to the development of the Virtual Piano Trainer. This paper will provide a proof of concept of whether such a system can train the upper extremities either unilaterally or bilaterally and combine proximal and distal training into a single activity or train each segment separately. Additionally, it presents information regarding the variability among stroke subjects and their responses to various rehabilitation interventions.

## Methods

### Development of the system

**Figure 1:**
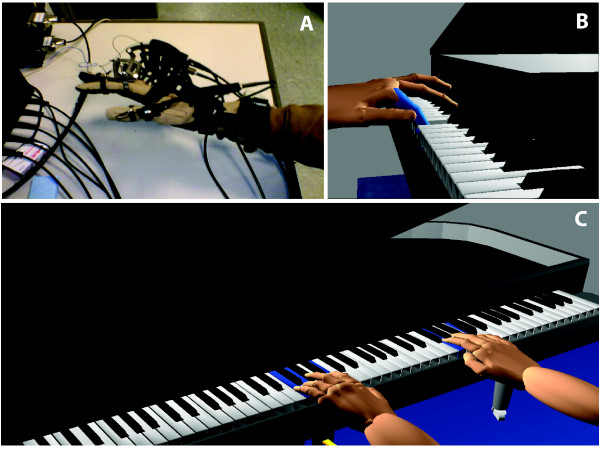
**Virtual Piano trainer**. A. CyberGrasp haptic device worn over a CyberGlove instrumented glove. B. Depiction of Virtual Key Press C. Piano Trainer Simulation; hands shown in a first person perspective.

The game architecture was designed so that various tracking mechanisms can be used to retrieve arm, hand, and finger movement data simultaneously. The system supports the use of a pair of CyberGloves (Immersion, USA), instrumented gloves for hand tracking. The Cyberglove weighs 220 grams. We combine this with a CyberGrasp (Immersion, USA) for haptic effects. The CyberGrasp device is a force-reflecting exoskeleton that fits over a CyberGlove data glove. It weighs 450 grams and can apply forces of various temporal profiles, up to 12 N to each finger (Fig. 1a). The Ascension Flock of Birds (FOB) (Ascension Technology Corporation, USA) is used for arm tracking.

The peripherals are connected to a PC (Pentium D 2.8 GHz, 1 GB RAM, 71.6 GB hard drive). The virtual environment was developed using Virtools software development package (Dassault Systemes, France) with the VR Pack plug-in which communicates with the open source VRPN (Virtual Reality Peripheral Network) server [12]. The VRPN server connects to the two instrumented gloves and the two FOB trackers through both standard and custom communication libraries. The glove and the FOB data are obtained at a baud rate of 115200 bits per second and translated through custom Virtools components. Position data collected by the Flock of Birds sensor is utilized to control movement of the avatar s arm in the virtual environment. The virtual arm must be positioned over the cued key on the 64 key virtual key board in order to elicit accurate virtual key-presses. Data from the glove and FOB sensor are coordinated in the sense that both sets of information affect avatar position. Delay time between movement and graphical updating is negligible (< 16 ms). Since this delay remains constant throughout the trials it does not affect measurements of movement duration or speed.

The virtual piano trainer presents a full keyboard with a complete range of single notes. A list of .wav files, each having the sound of a single note of the piano, are pre-loaded into a sound file array. The keyboard array of the virtual piano matches the sound file array, so whenever a key is successfully pressed, the corresponding sound file is played.

A configurable file allows the user to preset the key sequence which defines each song. A separate file allows the configuration of the song order, and duration for the exercise regimen. Songs or scales consisting of 5 to 10 notes are played in their entirety before participants are cued to begin playing individual notes. For each note, the current key and the corresponding finger which has to press the key are highlighted to cue the subjects as to which note should be played. The task of the subject is to then press the highlighted key with the highlighted finger. Upon successfully pressing the key by meeting the fractionation targets described below, the note will play and the next key will be lit.

The ability to visualize a representation of one s own hand moving through virtual space may strengthen a participant s feeling of being involved in an action and of attributing that action to themselves. This appears to be related to the degree of concordance between the intent of the movement, the participant s kinesthetic experience and the sensory feedback provided by the virtual environment. While utilizing the Piano Trainer, hand position and orientation as well as finger flexion and abduction is recorded in real time at 100 Hertz (HZ) and translated into three dimensional movements of the virtual hands which are shown on the screen in a first-person perspective (Fig. 1c). When key presses are achieved a visual representation of the key press is depicted through appropriate key rotation in order to maintain feedback integrity (Fig. 1b). The virtual environment was presented with non-immersive two-dimensional graphics.

### Calibration

Calibration in this study was accomplished by placing the hands into two different positions. The first position is as close to full extension and adduction of the fingers that can be attained and maintained passively. This step is approximated to angles of zero degrees at each joint. Each sensor reading for the finger flexion/extension angles is denoted by S_zero_. The second position is maximum finger flexion of all four fingers, which places each of the three finger joint angles at approximately 90 degrees. Each sensor reading in this case is denoted by S_ninety_. Having data from these two positions, the conversion factor between the readings of the glove sensors and the corresponding angles measured in degrees is found, and the joint angle measured in degrees can be calculated by using the formula:

(1)

Where A_actual _is the angle moved by the joint and S_actual _is the actual signal measured from the glove at the same instant. We have developed and tested several calibration algorithms that were more sophisticated than the one described here. However, for this particular simulation where interacting in virtual environment is driven by only four degrees of freedom of the hand (flexion/extension in the metacarpophalangeal joints of the four fingers), these lengthy calibration procedures were not required.

### Fractionation targets

**Figure 2:**
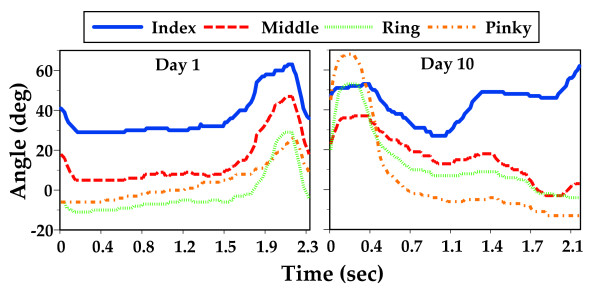
**Independent Finger Flexion**. *Left Panel*: Depiction of independent finger flexion preceding a virtual piano trainer intervention. Fingers are flexed as the subjects moves his hand to the cued key (first 1.5 seconds), then all four fingers flex as the subject attempts to press a piano key with his index finger. *Right Panel*: After nine days of training then, fingers are flexed initially during transport (first. 0.5 seconds) then the subject extends all four fingers (0.5 to 1.1 seconds) finally the non-cued fingers maintain flexion and the cued index finger flexes independently.

Fractionation is the ability to move each finger independently, measured as the flexion of the target finger in relation to the other fingers of the hand. Figure 2 depicts pre and post training differences in this ability for a representative subject. In the left panel all four fingers flex simultaneously as the subject attempts to strike a virtual key with his index finger. The right panel depicts the subject performing the same skill after nine days of training. Note the absence of flexion in the middle, ring and pinky fingers. In this study, fractionation score (FS) is calculated as the angle of the active finger s metacarpophalangeal (MCP) joint minus the MCP angle of the most flexed inactive finger. When the active finger flexes beyond the most flexed inactive finger the value is positive. When the inactive fingers are flexed beyond the active finger, the value is negative.

(2)

where i = {1,2,3}, A_active _is *joint angle of active finger *and A_non-active _(i) is *joint angle of the each non-active finger*.

The target fractionation score starts at 0° for each finger resulting in 8 fractionation targets. During an attempted key press, if the actual fractionation angle of a specific finger is equal to or becomes greater than the target fractionation, a successful piano key press will take place. After each trial (song) the system averages the fractionation achieved for each finger during that trial. If the average fractionation score is greater than 90% of the set target, the target fractionation will increase by 0.005 radians. If the average fractionation is less than 75% of the target, the target will decrease by the same amount. Therefore, successful achievement of the target fractionation in a previous trial increases the fractionation necessary to achieve a keystroke in a subsequent trial, and the target amount of fractionation decreases when the subject is unsuccessful.

A second requirement for a successful key press is having the active finger positioned correctly above the target piano key. The Virtools virtual environment has an inbuilt collision detection functionality which is used to detect the collision between the finger and the key in the virtual world. A successful key press is achieved and appropriate musical tone is generated only when the collision is detected at the appropriate key and the amount of finger flexion defined by the fractionation angle exceeds the targeted level.

### Adaptive algorithms

The criteria for all training tasks is to make the task challenging but not too frustrating, in order to make subjects work consistently and successfully. It is not known how best to accomplish this or what specific algorithm will facilitate the best outcomes. This system is flexible enough to accommodate different training paradigms or algorithms and we have used several varying algorithms in our training protocols. In the current study we tested two adaptive algorithms available to adjust target fractionation in response to participant performance.

Using Algorithm A, target fractionation starts at previous target level and decreases continuously until a key press occurs.

With F_actual _= actual fractionation and F_p _= previous target fractionation

(3)

T_total _= total time allowed for each key press which was predetermined to be 10 seconds in this study

Using Algorithm B diminution of target fractionation angle is delayed for six seconds and then decreases continuously until a key press occurs.

(4)

T_total _= total time allowed for each key press which was predetermined to be 10 seconds in this study

**Figure 3:**
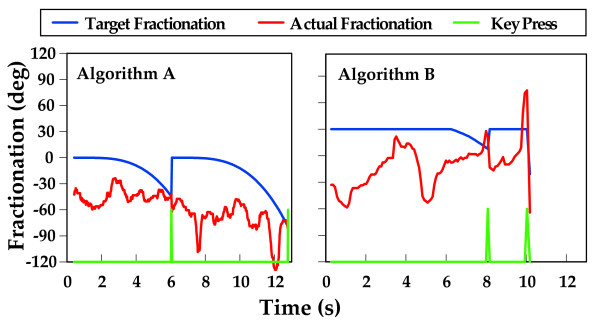
**Demonstration of Adaptive Algorithms A and B**. *Left Panel*: The blue line depicts target fractionation. The red line indicates the fractionation angles achieved by the subject during the attempted key presses. The green line indicates the timing of successful key presses. While training using Algorithm A, target fractionation decreases steadily until actual fractionation exceeds target fractionation. Key presses are unsuccessful because the actual fractionation (red line) does not meet the target fractionation (blue line) The green line indicates the timing of successful key presses. *Right Panel*: When training using Algorithm B diminution of target fractionation was delayed for six seconds, forcing the subject to attain a higher fractionation score to affect a successful key press.

Figure 3 is a graphic depiction of the application of algorithms A and B. The blue line depicts target fractionation. The red line indicates the fractionation angles achieved by the subject during the attempted key presses. The green line indicates the timing of successful key presses.

While training using Algorithm A (target fractionation starts at previous target level and decreases continuously until a key press occurs) this subject was able to achieve successful key presses at -45 and -63 degrees of fractionation. Whereas, when training using Algorithm B (diminution of target fractionation angle is delayed for six seconds), the subject was able to attain a higher fractionation score (30 degrees) to affect a successful key press.

### Haptic assistance

Simultaneous, synergistic flexion of all joints of the fingers, as well as difficulty with active finger extension following flexion is common sequelae of stroke. For subjects with these impairments, the CyberGrasp may be used to resist flexion of each of the fingers except for the active finger. We developed an interface for the CyberGrasp system and synchronized it with our virtual piano simulation running in Virtools. The piano simulation displays the active and non-active finger in the virtual environment, and triggers the CyberGrasp. A custom developed library for the Virtools environment controls the CyberGrasp. The force on the CyberGrasp is controlled such that the inactive fingers are given a much higher force than the active finger. As the subject improves his or her ability to flex their fingers one at a time, the haptic assistance can be gradually reduced. Tactile feedback can be provided when using the CyberGrasp. A small increase in resistance to finger flexion can be exerted on the distal phalanx of the active finger when a successful key press is achieved, providing the sensation of the finger contacting the piano key [13]. This force feedback is provided with a delay of < 32 ms, strengthening the sense of immersion in the VE.

### Proof of concept testing methods

We conducted a series of trials to establish the safety and viability of this system for the rehabilitation of hand and arm dysfunction due to stroke. Four subjects (mean age = 51.5 years) trained for ninety minutes per day using the virtual piano trainer completing between eight and nine sessions. Our subject sample varied across the spectrum of impairments from mild to moderate impairments, as per the Chedoke McMaster Stroke Assessment[14], they presented with minimal to moderate spasticity as measured by the Modified Ashworth Scale[15], and the time since stroke onset ranged from eleven months to seven years. We required ten degrees of active finger extension from resting position for inclusion in this study. (See Table 1). None of the subjects experienced adverse events or responses during or after training.

**Table 1:** Proof of concept study participant description

Subject	Age	Years Post CVA	Chedoke Arm	Chedoke Hand	Finger Flexor MAS
S1	44	8	5	3	2/4

S2	72	4	6	6	0/4

S3	44	1	6	6	2/4

S4	54	2	7	4	1+/4

Abbreviations:

CVA: cerebro-vascular accident, MAS: Modified Ashworth Scale

### Proof of concept testing results

**Figure 4:**
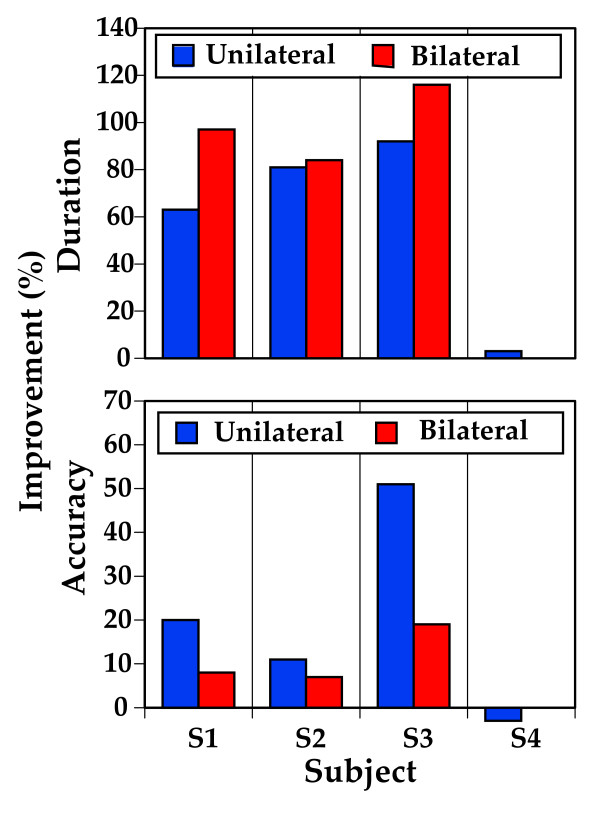
**Improvements in Accuracy and Task Duration**. Percent change in the time required to achieve successful key press (Duration, upper panel) and number of correct key presses (Accuracy, lower panel) are shown for each of the four subjects from the feasibility study following unilateral and bilateral piano training.

As a group, the four subjects improved in both key press duration and accuracy. Key press duration is a measure of the average time it takes to press a key after the note has been cued. Accuracy is measured by comparing the number of keys pressed correctly the first time to the total number of keys pressed. Higher values are achieved by striking fewer incorrect keys within the fixed number of cued keystrokes. Overall, subjects showed a 14% greater improvement in the time needed to press the correct key (duration) during the bilateral condition than in the unilateral condition. However there was an 8% larger increase in accuracy during the unilateral condition. The percent change made by individual subjects is displayed in Figure 4. Two of the four subjects showed more improvement in duration (Fig. 4, upper panel) 116%, and 97%, in the bimanual condition than in the unimanual condition. One subject performed similarly in both conditions (81% unilateral, 84% bilateral) and one subject did not improve their performance at all. In terms of key press accuracy (Fig. 4, lower panel) three of the four subjects performed better in the unilateral condition. This is to be expected as playing the piano with both hands is a very complex task and the goal of the training is not to necessarily improve bilateral piano playing but to use the disinhibition theoretically attained in the bilateral condition to facilitate activation in the lesioned cerebral hemisphere [2].

**Figure 5:**
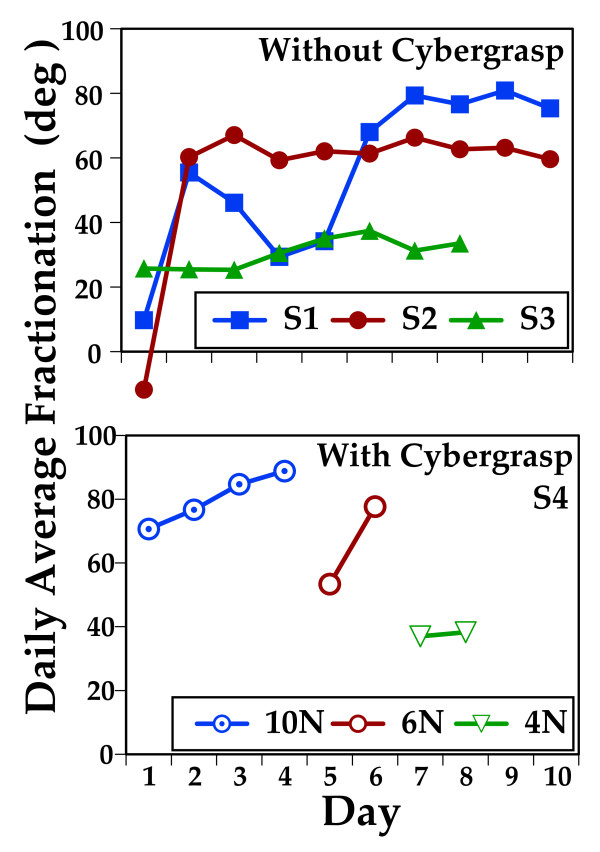
**Daily Average Fractionation Scores During Training**. *Upper Panel*: Fractionation Average daily fractionation for Subjects S1, S2 and S3 during 90 minute sessions using the Virtual Piano Trainer. Patterns of change vary. S1 increases average fractionation from 16 to 76 degrees. S2 improved from -15 to 63 degrees and S3 from 27 to 34 degrees. *Lower Panel*: Average daily fractionation for Subject S4, who used the CyberGrasp during training. His fractionations score improved over the first four days of training, with the CyberGrasp providing 10 Newtons of assistive force. On day five and six the assistive force was reduced to 6 Newtons. Fractionation diminishes initially but then improves. On training days seven and eight the force was further reduced to 4 Newtons of assistance. Fractionation diminishes again but then makes a small improvement.

Fractionation, the ability to move each finger individually is a construct critical to the manipulation of small objects during real world function. Three of the four subjects demonstrated improvements in their ability to flex their finger independently while performing this activity (Fig 5, upper panel). The percent change in the fractionation score ranged from 19% to 61%. Change score was calculated for each subject averaging first two day fractionation scores and comparing them to an average of the last two day fractionation scores [16]. Figure 5 (lower panel) presents average daily fractionation for Subject S4, who used the CyberGrasp during training. Without the CyberGrasp this subject could not achieve sufficient fractionation to utilize the Virtual Piano Trainer. On days one through four, the CyberGrasp provided 10 Newtons of force pulling his distal phalanges into extension. His fractionation improved from 70 to 90 degrees. On day five and six the assistive force was reduced to six Newtons. His ability to isolate his fingers diminished in response to this change but improved after further training from 54 to 80 degrees. On training days seven and eight the assistive force was further reduced to four Newtons, his ability to isolate his fingers again diminished but improved after further training with this amount of assistance from 36 to 38 degrees.

To test real world function we used two clinical measures, the Jebsen Test of Hand Function (JTHF) [17] and the Wolf Motor Function Test (WMFT) [18]. The two least impaired subjects improved their aggregate time on the JTHF (100 and 71 seconds respectively) which is consistent with our previous findings with this population [16,19]. One subject, who did not demonstrate progress (157 seconds at pre-test and 234 seconds at post-test), experienced difficulty with the checker stacking item of the JTHF. This problem accounted for all of the regression demonstrated in her score. Another subject was able to complete the small object lifting task from the JTHF after completing training. He could not do this previously. Two of the four subjects made modest improvements in WMFT aggregate score (9 and 4 seconds) and a third subject made significant improvements (59 seconds). The most impaired subject completed the checker stacking task from the WMFT at posttest which he was unable to perform during pre-testing. One subject who suffered from a weather-related increase in spasticity (thirty degree drop in temperature the day before post-testing), regressed on both tests. (134 seconds on the JTHF and 8 seconds on the WMFT). Despite these disappointing test scores this subject felt that he benefitted from treatment and volunteered to participate in future studies.

### Algorithm testing methods

Following the proof of concept study, we investigated the difference in the effects of two different assistance algorithms, A & B, on improving the ability to flex fingers independently while using the Virtual Piano Trainer. Two subjects trained for four days utilizing Algorithm A that decreased the target fractionation angle (as described in the methods) until the subject s attempted key press was successful. The two subjects trained four more days utilizing an Algorithm B that delayed this diminution of needed fractionation angle for six seconds, allowing the subject to make multiple attempts to press the key before the algorithm made the task easier. The two algorithm study subjects are not included in the proof of concept study analyses.

### Algorithm testing results

**Figure 6:**
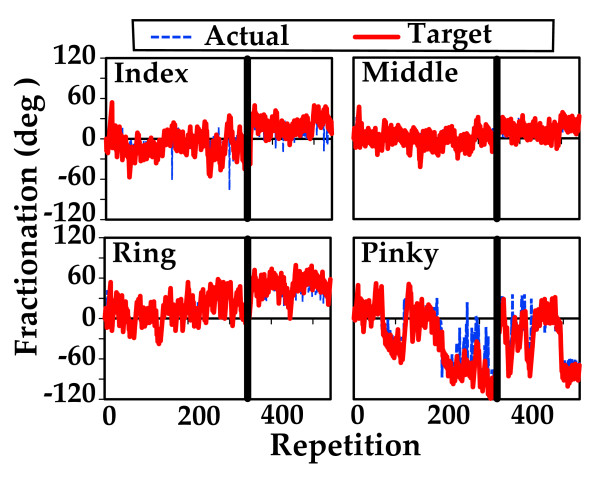
**Target and actual fractionation changes during training in subject S5**. The solid line depicts target fractionation and the dashed line depicts actual fractionation changes over two weeks of training for each of the four fingers for subject S5. The vertical line separates training with Algorithm A on the left and Algorithm B on the right (see Fig. 3). Minimal changes in fractionation were accomplished by this subject.

Subject S5, is a 78 year-old female 5 years post CVA with a Chedoke McMaster Hand Stage Classification of 6 [14]. Figure 6 presents the target fractionation and the actual fractionation changes during the entire training period for this subject, for four fingers. The red line is target fractionation. The blue line is actual fractionation. Training with algorithm A is before the horizontal black line and training with algorithm B is after the black line. Minimal changes in fractionation were made by this subject.

**Figure 7:**
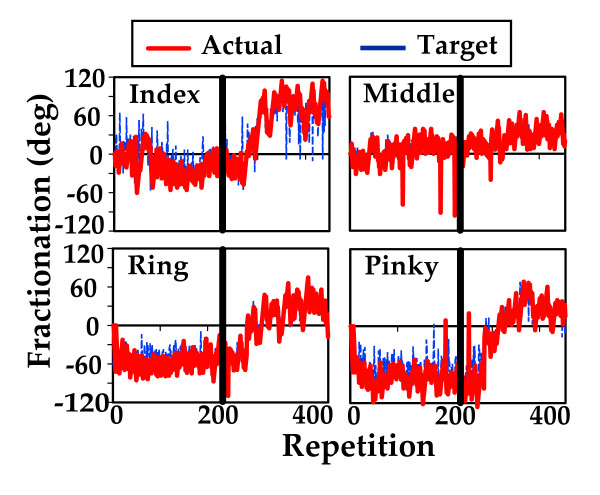
**Target and actual fractionation changes during training in subject S6**. This subject did not make gains when using algorithm A (on the left of the vertical line) but demonstrated dramatic improvements in his ability to isolate individual finger motion in three of his four fingers when using algorithm B (on the right of the vertical line). Subject demonstrated an increase in peak fractionation of 48 degrees for index finger, 165 degrees for middle finger and 72 degrees for pinky).

Figure 7 depicts the same variables for Subject S6; a 68 year-old male seven years post CVA with a Chedoke McMaster Hand Stage Classification of 4. This subject did not make gains when using algorithm A but demonstrated gradual improvements in his ability to isolate individual finger motion in three of his four fingers when using algorithm B (peak fractionation increases of 52 degrees for index finger, 20 degrees for middle finger, 80 degrees for his index finger and 63 degrees for his pinky). This might suggest that modifying the adaptive algorithm, could have an impact on the development of this skill and that impairment level might be a factor relevant to choosing the most effective algorithm for a given participant. This is an area of inquiry that will require extensive study in the future.

## Discussion

This initial study demonstrated that we have been able to develop a virtual-reality based system that models rehabilitation by; 1) creating a simulation that addresses specific hand impairments, 2) incorporates several input devices to accommodate patients with different levels of impairments, 3) provides unilateral and bilateral training and 4) combines training of the hand and arm into an integrated task-based simulation. This unique training modality is practical and accommodates and safely challenges subjects with a range of hand impairments evident post-stroke. Subjects were able to practice continuously for ninety minutes without ill effects. All of the subjects were able to interact with the VR simulation that combined hand and upper extremity motions, isolated finger activity and bimanual activities, even if they had difficulty with these types of activity in real world environments.

All four subjects were in the chronic phase post stroke and therefore considered to be neurologically stable. However, each of the subjects showed improvements in kinematic measures during their training activities and importantly, all of them demonstrated some improvement on elements of the clinical tests. The subjects in this proof of concept study presented with varying levels of spasticity, motor control, and active movement. Two of the subjects made significant gains and two did not, but these responses to treatment did not seem to correspond to impairment severities. Extensive study will be required to identify the impairments and patient characteristics that are best suited to this type of intervention in general, as well as the treatment scheduling and task parameters that suit each patient subgroup the best. However, we believe that the improvement in clinical performance that included some significant gains, in a population that would be presumed to be clinically stable, and not reporting recent gains in motor function suggests that this modality may be worthy of further study.

Modification of task difficulty to produce incremental changes in motor performance is an intervention approach associated with skill acquisition and neuroplasticity [20-22]. Adaptive algorithms that control robotic assistance or virtual task parameters are an extremely efficient method to accomplish this approach. The rapid changes in performance following algorithm adjustment experienced by one of the two subjects in our algorithm experiment suggest that manipulating the rate at which task parameters change may affect the rate at which a particular subject learned to perform a task. The algorithm experiment also suggests that the rate of task parameter change may interact with impairment level. The more impaired subject demonstrated larger performance changes after switching from algorithm A to algorithm B (task requirements more difficult). This experiment only offers a brief glimpse into the study of using adaptive algorithms to facilitate skill development. This finding may suggest that in VR training, more emphasis should be placed on individualizing treatment parameters as is done in real world therapy.

The flexibility afforded by the virtual piano trainer system will allow for the study of this concept in much greater depth.

The utilization of haptics to train individual fingers is a newer area of study. The combination of selective inhibition of abnormal finger flexion offered by the CyberGrasp with free arm motion described in this paper is unique. The Rutgers Master II allows for individual finger training and free arm motion but the pneumatic resistance offered by the system is generalized across all three fingers and the thumb and is constant [9]. Pneumatic and cable finger training systems described by Fischer allow for arm motion as well, but maintain a constant level of force throughout interventions [7]. The HandCARE system varies resistance from finger to finger and varies resistance during interventions but does not allow arm movement [23]. Kawasaki [8] developed a robot that trains individual finger flexion and extension in virtual environments utilizing robotic assistance that is controlled by the less impaired hand. This activity could allow for inhibition of the mass grasp pattern on individual fingers, but it is not coordinated with a simulation as in our system. To date, their pilot testing has not measured isolated finger flexion or real world function.

With the assistance of the CyberGrasp, one subject (S4) was able to use the system despite significant finger flexor dystonia and an inability to flex his fingers independently of each other. He was the only subject to utilize the CyberGrasp in this study. This subject made improvements in hand function after training as measured by the JTHF and elements of the WMFT. More impaired subjects, such as S4, have not demonstrated as much progress with previous iterations of our system [16,24]. This improvement may be due to the selective inhibition of only the inactive fingers.

## Conclusion

The design of suitable VR hand simulations are challenging due to the complexity of human hand function. However, this is a crucial area in need of systematic investigation, because the impact of even mild to moderate deficits in hand control in patients post stroke affect many activities of daily living, with detrimental consequences to social and work-related participation. Our current system allows for adjustments in point of view, bilateral and unimanual treatment modes, adaptive training algorithms and haptically rendered collisions. The rehabilitation literature describes promising results from these treatment elements, but controlled studies of the specific effects of these elements have not been done. This system allows for the study of multiple combinations of these virtual elements that may allow for quantification of the relative contribution of each, to the effectiveness of rehabilitation activities.

## Competing interests

The authors declare that they have no competing interests.

## Authors' contributions

SVA participated in the robotic/VR system design, study design, data collection, and data analysis and manuscript revision processes. GGF participated in the study design, subject recruitment, data collection, data analysis, initial manuscript preparation and manuscript revision. AM participated in the robotic/VR system design and data collection processes. QQ participated in the robotic/VR system design, data collection, data analysis and initial manuscript preparation. JL participated in the robotic/VR system design and manuscript revision processes. ASM participated in the robotic/VR system design, study design, data collection, data analysis, initial manuscript preparation and manuscript revision. All authors read and approved the final manuscript.
